# m5Cpred-XS: A New Method for Predicting RNA m5C Sites Based on XGBoost and SHAP

**DOI:** 10.3389/fgene.2022.853258

**Published:** 2022-03-30

**Authors:** Yinbo Liu, Yingying Shen, Hong Wang, Yong Zhang, Xiaolei Zhu

**Affiliations:** School of Sciences, Anhui Agricultural University, Hefei, China

**Keywords:** 5-cytosine-methylation, XGBoost, machine learning, shap, feature selection

## Abstract

As one of the most important post-transcriptional modifications of RNA, 5-cytosine-methylation (m5C) is reported to closely relate to many chemical reactions and biological functions in cells. Recently, several computational methods have been proposed for identifying m5C sites. However, the accuracy and efficiency are still not satisfactory. In this study, we proposed a new method, m5Cpred-XS, for predicting m5C sites of *H. sapiens*, *M. musculus*, and *A. thaliana*. First, the powerful SHAP method was used to select the optimal feature subset from seven different kinds of sequence-based features. Second, different machine learning algorithms were used to train the models. The results of five-fold cross-validation indicate that the model based on XGBoost achieved the highest prediction accuracy. Finally, our model was compared with other state-of-the-art models, which indicates that m5Cpred-XS is superior to other methods. Moreover, we deployed the model on a web server that can be accessed through http://m5cpred-xs.zhulab.org.cn/, and m5Cpred-XS is expected to be a useful tool for studying m5C sites.

## Introduction

RNA modification plays pivotal roles in many biological processes (Tang et al., 2001; Matzke et al., 2004; Xu et al., 2013; Jespersen et al., 2017; Xue Chen et al., 2020). Until now, about 170 types of RNA modifications have been discovered (Xuan et al., 2018), among which, 5-methylcytosine (m5C) is one of the most abundant post-transcriptional modifications (PTCM). In this modification, a methyl group is transferred to the fifth carbon atom of cytosine by RNA methyl-transferase (Jespersen et al., 2017). The m5C modification plays important roles in many biochemical reactions (Catania and Fairweather 1991; Fasolino et al., 2017; Yang et al., 2017; He et al., 2020; Xue MiaoMiao et al., 2020; Zhang et al., 2020), such as the pathogenesis of various cancers (He et al., 2020; Xue MiaoMiao et al., 2020; Zhang et al., 2020), rRNA assembly (Zhang et al., 2020), and cellular aging (Catania and Fairweather 1991), etc. Thus, it is meaningful to pinpoint m5C sites in RNA sequences.

Several experimental methods have been developed to identify m5C sites, including miCLIP-seq (Hussain et al., 2013), Aza-IP-seq (Khoddami and Cairns 2013), bisulfite sequencing (Agris 2008; Schaefer et al., 2010), and m5C-RIP-seq (Khoddami et al., 2019). However, these methods have their own shortcomings (Fu et al., 2012). For example, bisulfite sequencing cannot detect m5C sites in low-abundance RNA. Moreover, these existing experimental methods are time-consuming and expensive. In recent years, with the development of computer technology, several computational methods, especially those machine-learning based methods, have been developed for RNA m5C site identification (Feng et al., 2016; Qiu et al., 2017; Sabooh et al., 2018; Zhang et al., 2018).

The computational methods are mainly classified into two categories: random forest (RF)-based models and support vector machine (SVM)-based models. Based on RF, Qiu et al. (2017) proposed iRNAm5C-PseDNC based on pseudo dinucleotide composition (PseDNC) feature encoding, and Li et al. (2018) constructed RNAm5Cfinder by using mononucleotide binary encoding (MNBE) to encode the RNA sequences. Based on these two feature encodings and K-tuple nucleotide frequency component (KNFC), Song et al. (2018) developed a predictor named PEA-m5C. By using SVM as the classifier, Feng et al. (2016) developed m5C-PseDNC based on features of PseDNC. Fang et al. (2019) built RNAm5CPred based on the features of PseDNC, KNFC, and MNBE. By integrating multiple SVM methods, Zhang et al. (2018) developed an ensemble model, m5C-HPCR, by incorporating different physical–chemical properties into PseDNC. Chen Xiao et al. (2020) proposed another SVM-based model, m5CPred-SVM, which uses six sequence-based features, including k-nucleotide frequency (KNF), K-spaced nucleotide pair frequency (KSNPF), position-specific nucleotide propensity (PSNP), K-spaced position-specific dinucleotide propensity (KSPSDP), PseDNC, and chemical property with density (CPD).

As mentioned above, different kinds of features have been generated for predicting m5C sites, and the dimension of these features can be very high; however, not all the features are relevant for building machine learning models. Moreover, the features with ultrahigh dimensions also pose a great challenge to computer performance (Li et al., 2021). Selecting the optimal feature subset by appropriate feature selection methods can not only improve the accuracy of the prediction model, but also effectively reduce the huge computing power required for model training.

Recently, different feature selection methods have been used in developing models for predicting the RNA modification sites. Wang et al. (2018) used a minimum redundancy maximum (mRMR) correlation algorithm to select discriminative features from the features encoded based on RNA sequences. Sabooh et al. (2018) developed a new computational method pm5CS-Comp-mRMR by also using mRMR for selecting the discriminate features. Furthermore, Visentini et al. (2016) first sorted the features according to the F-score obtained in the eXtreme gradient boosting (XGBoost) (Chen, 2016) package and then selected the top 50 features based on the incremental feature selection (IFS) strategy as the optimal feature subset. To reduce the dimension of features, Chai et al. (2021a) proposed an efficient m5C sites prediction approach, Staem5, based on features selected by F-score. The SHapley Additive ExPlanations (SHAP) (Wang and Gribskov 2019; Bi et al., 2020) method, which can interpret the importance of features, is another effective method for selecting relevant features. The method was also used in several recent works (Bi et al., 2020; Pathy et al., 2020; Effrosynidis and Arampatzis 2021).

In this study, we established a new method to predict m5C sites by using XGBoost based on features selected by SHAP. We named this method m5Cpred_XS, which can be used to predict m5C sites in multiple species. Extensive experiments demonstrated that the proposed predictor, m5Cpred_XS, outperformed other existing prediction methods. Finally, a web server (http://m5cpred-xs.zhulab.org.cn/) was deployed for the users.

## Materials and Methods

### Overall Framework of m5Cpred_XS

**FIGURE 1:**
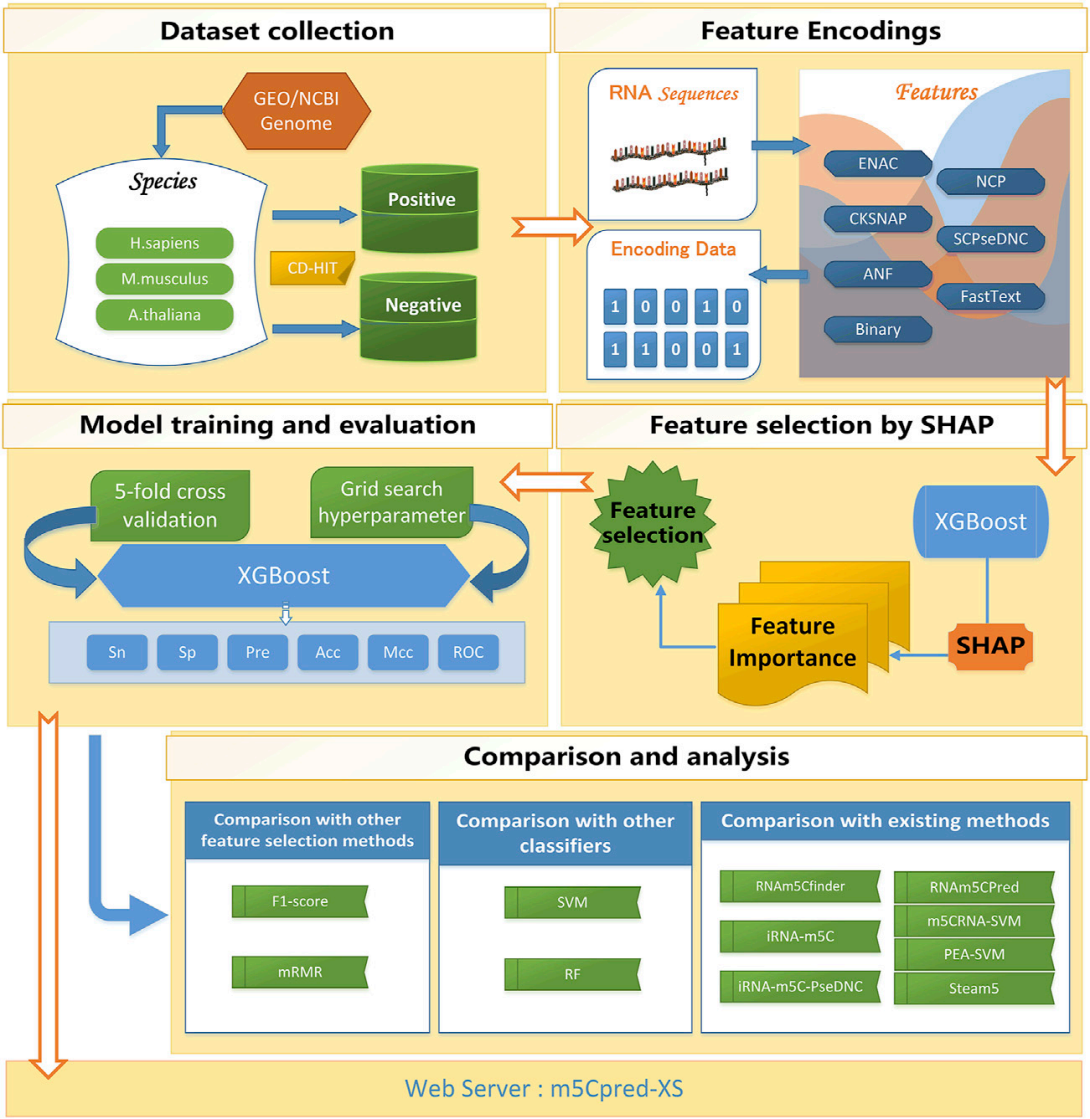
The flowchart of m5Cpred_XS.

For building our model reasonably, we conducted our study in six steps. I) A benchmark data set was collected. The benchmark data set was divided into the training set and the independent test set. II) The features were extracted from RNA sequences. III) The SHAP-based feature selection was carried out to select the optimal feature subset. IV) The XGBoost was used to train the model. V) The comparison and analysis of different models was conducted. VI) A web server for predicting m5C sites was developed for the community. The overall flow chart of our study is shown in Figure 1.

### Benchmark Data Sets

For fair comparison, we used the same data sets as in Chen Xiao et al. (2020). In their work, they collected data for three species: *H. sapiens*, *M. musculus,* and *A. thaliana*. As shown in Table 1, the data sets contain 269, 5563, and 6289 positive samples for the three species, respectively, and the numbers of negative samples are the same as positive samples. The positive samples of *H. sapiens, M. musculus*, and *A. thaliana* were collected from the work of Yang et al. (2017), Khoddami et al. (2019), and Cui et al. (2017), respectively. For the details about how the data sets were obtained, please refer to Chen Xiao et al. (2020).

**TABLE 1:** Training and test data sets of three species.

Datasets^a^	Length (bp)	Positive subset	Negativity subset
H_train	41	200	200
H_test	41	69	69
M_train	41	4,563	4,563
M_test	41	1,000	1,000
A_train	41	5,289	5,289
A_test	41	1,000	1,000

a H, M and H, M, A represent *H. sapiens*, *M. musculus* and *A. thaliana*, respectively.

To build and evaluate the models, the benchmark data sets were divided into two parts: the training data sets and the independent test sets. The training data sets were used for the model construction, cross-validation, and the determination of the hyperparameters of machine learning algorithms, whereas the independent test sets were used for testing the prediction performance and generalization ability of the models. For *A. thaliana*, 1000 positive and 1000 negative samples were randomly selected from the data set as the independent test data set, and the remaining 5298 positive and 5298 negative samples were selected as the training data set. Similarly, 1000 positive and 1000 negative samples from *M. musculus*’ benchmark data set were selected as the independent test set, and the remaining 4563 positive and 4563 negative samples were selected as the training data set. For *H. sapiens*, 69 positive and 69 negative samples were randomly selected as the independent test set, and the remaining 200 positive and 200 negative samples were selected as the training data set. The specific partitioning of the data sets is shown in Table 1.

For each RNA segment, it can be expressed in the following form:

$$R_{\lambda }\left(C\right)=N_{-\lambda }N_{-\left(\lambda -1\right)}\ldots N_{-1}CN_{1}\ldots N_{\lambda -1}N_{\lambda }.$$

In this formula, $N_{\lambda }$ and $N_{-\lambda }$ represent the downstream and upstream nucleotide with cytosine (C) at the center, respectively. Previous studies (Hussain et al., 2013; Khoddami and Cairns 2013; Qiu et al., 2017; Sabooh et al., 2018; Zhang et al., 2018; Khoddami et al., 2019) show that the performance is better when λ is set to 20. Therefore, in this study, we also set λ=20 , which means that all the RNA segments have a length of 41 bp.

### Feature Encoding Extraction

#### Enhanced Nucleic Acid Composition

ENAC encoding (Ahmad and Shatabda 2019) is used for feature extraction in equal-length RNA sequences. It first determines a fixed length window, and then the window is slid from the 5-terminal to the 3-terminal of the RNA segment without interval. The features of ENAC are expressed as follows (Han et al., 2019):

$$V=\left[\frac{N_{A,\mathrm{wi}n_{1}}}{S},\frac{N_{c,\mathrm{wi}n_{1}}}{S},\frac{N_{G,\mathrm{wi}n_{1}}}{S},\frac{N_{U,\mathrm{wi}n_{1}}}{S},\frac{N_{A,\mathrm{wi}n_{2}}}{S},\frac{N_{C,\mathrm{wi}n_{2}}}{S},\ldots ,\frac{N_{C,\mathrm{wi}n_{L-S+1}}}{S},\frac{N_{G,\mathrm{wi}n_{L-S+1}}}{S},\frac{N_{U,\mathrm{wi}n_{L-S+1}}}{S}\right].$$

In this formula, S represents the size of the sliding window, and $N_{t,r}$ represents the number of nucleotide t in this window r (r=1,2,…,L−S+1,t∈{A,C,G,U}) . In this paper, the value of S is set to five; thus, the dimension of ENAC is 148.

#### The Composition of K-Spaced Nucleic Acid Pairs

The CKSNAP feature encoding scheme (Cui et al., 2017; Ju and Wang 2020) is based on the frequency of k-spaced nucleotide pairs (k = 0, 1, 2, 3, 4, 5). For example, when k = 1, the nucleotide pairs corresponding to k-spaced 16 possible nucleotide pairs (i.e., “A∗A″, “A∗C″, “A∗G″, …, “C∗G″, “G∗A″, …, “G∗C″, “U∗U″, “U∗A″, “U∗C″, “U∗G″), CKSNAP can be expressed as the following formula:

$${\left(\frac{N_{\mathrm{A*A}}}{N_{\mathrm{total}}},\frac{N_{\mathrm{A*C}}}{N_{\mathrm{total}}},\frac{N_{\mathrm{A*G}}}{N_{\mathrm{total}}},\ldots ,\frac{N_{\mathrm{T*T}}}{N_{\mathrm{total}}}\right)}_{\mathrm{16}},$$

where ∗ represents k arbitrary nucleotides, and $N_{A*A}$ represents the number of nucleotide pairs A∗A appearing in the entire RNA sequence. $N_{total}$ represents the total number of nucleotide pairs appearing in the RNA sequence with the interval k. A total number of 96 (16∗6) dimensional features were generated by CKSNAP encoding.

#### Accumulated Nucleotide Frequency

ANF, also known as nucleotide density (ND), fully considers the distribution and nucleotide frequency information of each nucleotide in the RNA sequence (Chen Zhen, et al., 2020). The density of a nucleotide $n_{i}$ at i position in RNA sequence can be expressed as follows:

$$d_{i}=\frac{1}{i}\sum_{j=1}^{i}f\left(S_{j}\right),f\left(q\right)=\{\begin{array}{c} 1,n_{i}=q\;\;\;\;\;\;\;\;\;\;\\ 0,\mathrm{otherwise}, \end{array}$$

where $S_{j}$ represents the type of nucleotide at the sequence position j . For example, an RNA sequence ‘AUCUCAUGAG,’ the densities of A at positions 1, 6, and 9 can be expressed as 1.00 (1/1), 0.33 (2/6), and 0.33 (3/9). The densities of U at positions 2 and 4 are 0.50 (1/2), 0.50 (2/4), respectively. In this way, the whole RNA sequence can be expressed as (1.00.0.50.0.33.0.50, 0.20.0.33.0.43.0.13.0.33.0.20). ANF produces 41 dimensional features for a 41-bp RNA sequence.

#### Nucleotide Chemical Property

Adenine (A), guanine (G), cytosine (C), and uracil (U) are the four types of nucleotides in RNA, each of which has unique chemical properties and physical structure. According to different chemical properties, these four nucleotides can be divided into three categories (Chen et al., 2016). The details are shown in Table 2.

**TABLE 2:** Chemical structure of each nucleotide.

Chemical property	Class	Nucleotides
Ring Structure	Purine	A, G
	Pyrimidine	C, U
Functional Group	Amino	A, C
	Keto	G, U
Hydrogen Bond	Strong	C, G
	Weak	A, U

Based on the three types of chemical properties, A, C, U, and G can be expressed as (1, 1, 1), (0, 1, 0), (1, 0, 0), and (0, 0, 1), respectively. The feature dimension generated by NCP is 123.

#### Binary Encoding

The method of using a four-dimensional binary vector to encode the nucleotide is called the binary encoding scheme (Foster et al., 2003) by which A, C, G, and U are encoded as (1, 0, 0, 0), (0, 1, 0, 0), (0, 0, 1, 0), and (0, 0, 0, 1), respectively. Thus, we obtained a 164-dimensional feature vector for an RNA segment containing 41 nucleotides.

#### Series Correlation Pseudo Dinucleotide Composition

The expression of SCPseDNC (Chen et al., 2014) coding is as follows:

$$D={\left[d_{1},d_{2},d_{3},\ldots d_{\mathrm{16}},d_{\mathrm{16}+1},\ldots ,d_{\mathrm{16}+\lambda },\ldots ,d_{\mathrm{16}+\lambda \Lambda }\right]}^{T},$$

where $d_{k}$ represents

$$d_{k}=\{\begin{array}{c} \frac{f_{k}}{\sum_{i=1}^{\mathrm{16}}f_{i}+w\sum_{j=1}^{\lambda }\theta_{j}}\;\;\;\;\;\;\;\left(1\leq k\leq \mathrm{16}\right)\\ \frac{w\theta_{k-\mathrm{16}}}{\sum_{i=1}^{\mathrm{16}}f_{i}+w\sum_{j=1}^{\lambda \Lambda }\theta_{j}}\;\;\;\;\;\;\left(\mathrm{17}\leq k\leq \mathrm{16}+\lambda \Lambda \right) \end{array}.$$

Here, $f_{k}\left(k=1,2,\ldots ,16\right)$ is the standardized occurrence frequency of the 16 types of dinucleotides in a sequence, λ represents the highest counted rank (or tie) of the correlation along the nucleotide sequence, w is the weight, which ranges from zero to one, and Λ is the six physicochemical indices, including ‘Roll (RNA)', ‘Rise (RNA)', ‘Shift (RNA)', ‘Twist (RNA)', ‘Slide (RNA)' and ‘Tilt (RNA)'. $\theta_{j}\;\left(j=1,2,\ldots ,\lambda \right)$ is the j -tier correlation factor, defined as follows:

$$\{\begin{array}{c} \theta_{1}=\frac{1}{L-3}\sum_{i=1}^{L-3}j_{i,i+1}^{1}\\ \theta_{2}=\frac{1}{L-3}\sum_{i=1}^{L-3}j_{i,i+1}^{2}\\ \ldots \ldots \\ \theta_{\Lambda }=\frac{1}{L-3}\sum_{i=1}^{L-3}j_{i,i+1}^{\Lambda }\left(\lambda <L-2\right)\\ \ldots \ldots \\ \theta_{\lambda \Lambda -1}=\frac{1}{L-\lambda -2}\sum_{i=1}^{L-\lambda -2}j_{i,i+1}^{\Lambda -1}\\ \theta_{\lambda \Lambda }=\frac{1}{L-\lambda -2}\sum_{i=1}^{L-\lambda -2}j_{i,i+1}^{\Lambda } \end{array},$$

where the correlation function $j_{i,i+k}^{\varsigma }$ is defined as

$$\{\begin{array}{c} J_{i,i+m}^{\varsigma }=P_{\varsigma }\left(R_{i}R_{i+1}\right)P_{\varsigma }\left(R_{i+m}R_{i+m+1}\right)\;\\ \varsigma =\mathrm{1,2},\ldots ,\Lambda ;m=\mathrm{1,2},\ldots ,\lambda ;i=\mathrm{1,2},\ldots ,L-\lambda -2 \end{array},$$

where ς is the number of physicochemical indices. $P_{\varsigma }\left(R_{i}R_{i+1}\right)$ is the value of the ς‐th physical and chemical index of the i -dinucleotide $R_{i}R_{i+1}$ . $P_{\varsigma }\left(R_{i+m}R_{i+m+1}\right)$ refers to the value of the ς‐th physical and chemical index of the i+m -dinucleotide $R_{i+m}R_{i+m+1}$ . In this paper, we set λ=20 and w=0.9 to generate a 136-dimensional feature vector.

#### Word2Vec by FastText

FastText is a natural language model released by Facebook (Joulin et al., 2017). By considering the RNA segments as sentences, we used the FastText program to build a word2vec model and then to encode the RNA segments as word vectors. Both skipgram and cbow models can be trained in FastText; we, thus, trained a cbow model to generate word embeddings for RNA segments. A total of 100-dimensional feature data was generated by using FastText.

### Feature Selection

Feature selection is an important step in building effective machine learning models when high-dimensional features were generated. In this study, three different feature selection methods were employed to select the optimal feature subsets. As one of the frameworks for explaining the prediction model, the SHAP algorithm was proposed to characterize feature importance and assess feature behavior (Swann et al., 2011). The contribution of each feature can be evaluated by the SHAP value, which is calculated by

$$\Gamma_{i}=\sum_{S\subseteq F,\left\{i\right\}}\left(\left|S\right|!\left(\left|F\right|-\left|S\right|\mathrm{-1}\right)\mathrm{!/}\left|F\right|!\right)\left[f_{S\cup \left\{i\right\}}\left(x_{S\cup \left\{i\right\}}\right)-f_{S}\left(x_{S}\right)\right],$$

where ${\text{Γ}}_{i}$ represents the importance score of the feature i , F denotes the set of all features, and S expresses all feature subsets obtained from F without feature i . The predictive results of the two models based on $f_{S\cup \left\{i\right\}}$ and $f_{S}$ were compared with the current input $f_{S\cup \left\{i\right\}}\left(x_{S\cup \left\{i\right\}}\right)-f_{S}\left(x_{S}\right)$ , where $x_{S}$ represents the values of the input features in the set S . To estimate ${\text{Γ}}_{i}$ based on the $2^{\left|F\right|}$ difference, the SHAP method approximates the Shapley value by performing Shapley sampling or Shapley quantitative influence.

The F-score (Polat and Guenes 2009) is another feature selection method that measures the discriminatory ability of two sets of real values. The F-score value of each feature in the data set can be calculated by the following equation:

$$F_{i}=\frac{{\left({\bar{x}}_{i}^{\left(+\right)}-{\bar{x}}_{i}\right)}^{2}+{\left({\bar{x}}_{i}^{\left(-\right)}-{\bar{x}}_{i}\right)}^{2}}{\frac{1}{n_{+}-1}\sum_{k=1}^{n_{+}}{\left(x_{k,i}^{\left(+\right)}-{\bar{x}}_{i}^{\left(+\right)}\right)}^{2}+\frac{1}{n_{-}-1}\sum_{k=1}^{n_{-}}{\left(x_{k,i}^{\left(-\right)}-{\bar{x}}_{i}^{\left(-\right)}\right)}^{2}},$$

where $F_{i}$ represents the F-score value of the ith feature; ${\bar{x}}_{i}$ , ${\bar{x}}_{i}^{\left(+\right)}$ , ${\bar{x}}_{i}^{\left(-\right)}$ are the average of the ith feature of all, positive, and negative samples of the data set, respectively; $n_{+}$ and $n_{-}$ mean the numbers of positive and negative samples in the data set, respectively; $x_{k,i}^{\left(+\right)}$ is the ith feature of the kth positive sample; and $x_{k,i}^{\left(-\right)}$ is the ith feature of the kth negative sample. Thus, the numerator means the variance between means of the positive and negative samples, and the denominator represents the sum of variances of positive and negative samples. The larger the F-score, the more likely this feature is to be more discriminative.

The third feature selection method used in this study is maximum relevance minimum redundancy (mRMR), which was developed by Peng et al. (Hanchuan et al., 2005). In this method, mutual information (MI) is used to evaluate the relationships among the features and the labels, and the goal of the method is to identify features that can maximize the relevance between features and labels and simultaneously minimize the relevance between the features. The following equation is used to select features recursively:

$$\underset{f_{j}\in \Omega_{r}}{\max}\left[I\left(f_{j},l\right)-\frac{1}{\left|\Omega_{s}\right|}\sum_{f_{i}\in \Omega_{s}}I\left(f_{j},f_{i}\right)\right],$$

where ${\text{Ω}}_{s}$ represents the subset with selected features and ${\text{Ω}}_{r}$ represents the subset of remaining features; $f_{j}$ and $f_{i}$ represent the features in ${\text{Ω}}_{s}$ and ${\text{Ω}}_{r}$ , respectively; l represents the label vector; I(x,y) means the mutual information between vector x and y , which can be calculated as follows:

$$I\left(x,y\right)=\iint p\left(x,y\right)\log\frac{p\left(x,y\right)}{p\left(x\right)p\left(y\right)}\mathrm{dxdy},$$

where p(x,y) is the joint probabilistic density and p(x) , p(y) are the marginal probabilistic densities.

### Classifier

The XGBoost was a distributed gradient enhancement library that was widely used in classification scenarios (Ji et al., 2019; Zhao et al., 2019; Ding et al., 2020; Samat et al., 2020). It has many advantages, such as flexibility, efficiency, and portability. The basic principle of this algorithm is to assign quantitative weight to each leaf node of a series of decision trees. The parallel enhanced trees are provided by XGBoost. This algorithm has very good ability to process sparse and high-dimensional data, and it also inherits the high accuracy of the original boosting algorithm. Some researchers apply this algorithm in bioinformatics, such as the prediction of m6A (Qiang et al., 2018; Zhao et al., 2019) and m7G sites (Bi et al., 2020). In this paper, we used a python package to build the XGBoost model and used a grid search method to optimize hyperparameters, max_depth, learning_rate, and n_estimators. The ranges of these three hyperparameters are (2, 4, 6, 8,10, 12, 14.16), (0.005, 0.01, 0.02, 0.05, 0.1), and (1,600,1800,2000, 2,200, 2,400, 2,600, 2,800), respectively. Finally, we obtained different optimal hyperparameters for different species. The optimal hyperparameters for three species are shown in Table 3.

**TABLE 3:** The optimal hyperparameters of XGBoost for three species.

Species	learning_rate	max_depth	n_estimators
*H. sapiens*	0.05	2	2000
*M. musculus*	0.02	6	2,600
*A. thaliana*	0.01	16	1800

### Evaluation Criteria

Cross-validation is often used to evaluate the performance and generalization ability of machine learning models. In this paper, five-fold cross-validation was used to evaluate the models, and the random sampling method was used to divide the training data set into five subsets with very close data volume (Fushiki 2011). In each training, one of the five subsets was used as validation data set, and the other four were used for training the model. Thus, a total of five m5C site prediction models were obtained. Finally, the prediction results of these five models were evaluated, and the five evaluation values were averaged as the ultimate evaluation indices. Similarly, this five-fold cross-validation was also adopted for hyperparameter selection, algorithm comparison, etc.

Different evaluation metrics are used in bioinformatics classification. In this study, we selected the accuracy (Acc), sensitivity (Sen), specificity (Spe), precision (Pre), Matthews correlation coefficient (Mcc), and F1-score as the main evaluation metrics (Zhang et al., 2019; Lv et al., 2020). Counts of true positive, true negative, false positive, and false negative predictions were recorded as TP, TN, FP, and FN, respectively. Thus, the six metrics can be represented as follows:

$$\{\begin{array}{c} \mathrm{Sen}=\frac{\mathrm{TP}}{\mathrm{TP}+\mathrm{FN}}\\ \begin{array}{c} \mathrm{Spe}=\frac{\mathrm{TN}}{\mathrm{TN}+\mathrm{FP}}\\ \mathrm{Pre}=\frac{\mathrm{TP}}{\mathrm{TP}+\mathrm{FP}} \end{array}\\ \begin{array}{c} \mathrm{Acc}=\frac{\mathrm{TP}+\mathrm{TN}}{\mathrm{TP}+\mathrm{FP}+\mathrm{TN}+\mathrm{FN}}\\ \mathrm{Mcc}=\frac{\mathrm{TN*TP}-\mathrm{FN*FP}}{\sqrt{\left(\mathrm{TP}+\mathrm{FP}\right)\left(\mathrm{TP}+\mathrm{FN}\right)\left(\mathrm{TN}+\mathrm{FP}\right)\left(\mathrm{TN}+\mathrm{FN}\right)}}\\ F1=\frac{2\mathrm{*TP}}{\left(2\mathrm{*TP}+\mathrm{FP}+\mathrm{FN}\right)} \end{array} \end{array}$$

In addition to the above evaluation indicators, the precision recall curve (PRC curve) (Keilwagen et al., 2014; Saito and Rehmsmeier 2017) and receiver operating characteristic curve (ROC curve) (Fawcett 2006; Li et al., 2019) were also used to evaluate the model. These two curves have the ability to evaluate the prediction performance of the proposed method in the whole decision value range, and the areas under the curves (AUPRC and AUROC) are often used to quantify the performance of the models. We quantify the performance of the model by plotting these two kinds of curves and calculating the areas under the ROC and PRC curves.

## Results

### Models Based on Features Selected by SHAP

**FIGURE 2:**
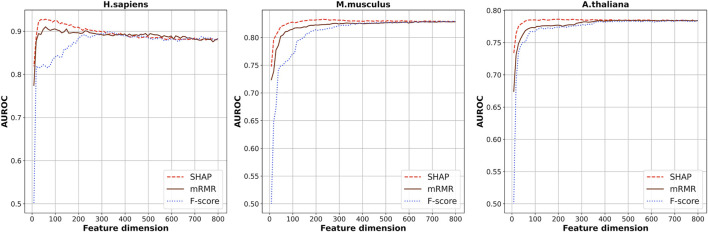
The cross-validation AUROC values of models based on the top *n* features selected by SHAP, mRMR, and f-score.

Seven kinds of features were generated from the RNA segments of the three species of which the dimension is 808 in total. Considering the redundancy between the features, SHAP was used to select the optimal feature subsets by which the scores of importance of the 808-dimensional features were calculated based on XGBoost ensemble algorithm. Figure 2 shows the cross-validation AUROC values of models based on the top *n* features. The highest AUROCs were obtained when the top 48, 228, and 208 features were used for *H. sapiens*, *M. musculus*, and *A. thaliana*, respectively. The corresponding AUROC values are 0.935, 0.834, and 0.787, for the three species, respectively.

In addition, Table 4 shows all the evaluation metrics for the models based on features selected by SHAP and the models based on the original 808 features. It indicates that the models based on features selected by SHAP achieved higher values than the model based on the original 808 features for most of the metrics, which demonstrates the advantages of using SHAP for feature selection.

**TABLE 4:** The five-fold cross-validation results for models based on features selected by SHAP or the original 808 features.

Species	Feature used	Pre (%)	Sp (%)	Sn (%)	Acc (%)	F1	MCC	AUROC
*H.sapiens*	Features selected by SHAP	**83.2**	82.0	**89.0**	**85.5**	**0.860**	**0.712**	**0.935**
	808 features	78.9	78.5	80.5	79.5	0.797	0.590	0.873
*M.musculus*	Features selected by SHAP	**75.1**	**74.9**	75.6	**75.3**	**0.754**	**0.505**	**0.834**
	808 features	74.7	74.2	**76.1**	75.1	**0.754**	0.503	0.831
*A.thaliana*	Features selected by SHAP	**74.8**	76.9	**68.5**	**72.7**	**0.715**	**0.456**	**0.787**
	808 features	73.6	75.9	67.3	71.6	0.703	0.434	0.779

### Comparison With Other Feature Selection Methods

Besides this, another two kinds of feature-selection methods, F-score (Polat and Guenes 2009) and mRMR (Li et al., 2017; Bugata and Drotar 2020), were also used to select the optimal feature subsets. The cross-validation AUROCs of the models based on the top *n* features selected by these two methods are also plotted in Figure 2. As shown in Figure 2, generally, the models based on features selected by SHAP are superior to the models based on features selected by the other two methods. Thus, we used the feature subsets selected by SHAP as the optimal feature subsets.

### Models Based on Different Classifiers

To verify the effectiveness of the XGBoost algorithm in m5C site prediction, two other learning algorithms, random forests (Biau 2012; Ziegler and Konig 2014; Li et al., 2018) and support vector machine (Boopathi et al., 2019; Chen et al., 2019; Liu et al., 2020), were also used to build models based on the optimal feature subsets selected by SHAP. The hyperparameters of RF and SVM were also optimized by grid search.

**FIGURE 3:**
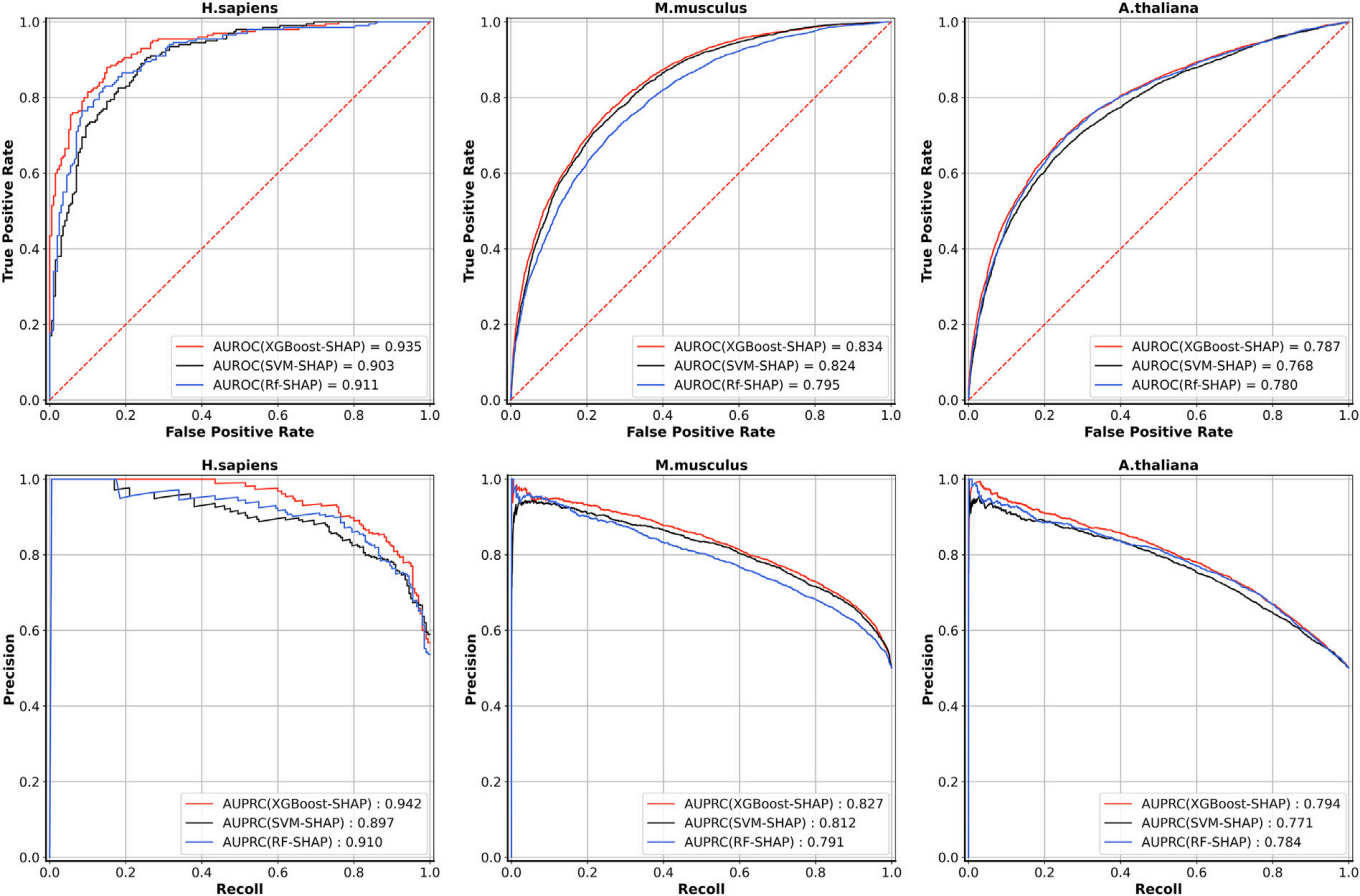
The ROC curves and PRC curves of five-fold cross-validation results based on three learning algorithms for the three species.

Table 5 shows the five-fold cross-validation performances for the models based on the three different learning algorithms. For *A. thaliana*, the AUROC value of the model based on XGBoost is 0.787, which is higher than the models based on RF (0.780) and SVM (0.768). For *M. musculus*, the AUROC value of the model based on XGBoost is 0.834, which is also higher than the models based on RF (0.795) and SVM (0.824). For *H. sapiens*, the AUROC value of the model based on XGBoost is 0.935, which is also higher than the models based on RF (0.911) and SVM (0.903). The ROC and PRC curves for three species are shown in Figure 3. As shown in Figure 3, for *H. sapiens*, the AUPRC of the model based on XGBoost is 0.942, which is higher than the models based on RF (0.910) and SVM (0.897). Similarly, for *A. thaliana*, the AUPRC of the model based on XGBoost is 0.794, which is higher than that based on RF (0.784) and SVM (0.771). In addition, for *M. musculus*, the AUPRC of the model based on XGBoost is 0.827, which is higher than the models based on SVM (0.812) and RF (0.791). Thus, the models built by using XGBoost were selected as our final models.

**TABLE 5:** The five-fold cross-validation performance of models built based on different classifiers with the features selected by SHAP.

Species	Classifiers	Pre (%)	Sp (%)	Sn (%)	Acc (%)	F1	MCC	AUROC
*H. sapiens*	RF	82.8	**82.5**	84.5	83.5	0.837	0.670	0.911
	SVM	79.9	79.0	83.5	81.3	0.817	0.626	0.903
	XGBoost	**83.2**	82.0	**89.0**	**85.5**	**0.860**	**0.712**	**0.935**
*M. musculus*	RF	70.7	69.2	74.4	71.8	0.725	0.437	0.795
	SVM	73.5	72.6	76.0	74.3	0.747	0.487	0.824
	XGBoost	**75.1**	**74.9**	75.6	**75.3**	**0.754**	**0.505**	**0.834**
*A. thaliana*	RF	75.1	**78.4**	65.3	71.8	0.699	0.441	0.780
	SVM	74.2	78.2	62.9	70.5	0.681	0.416	0.768
	XGBoost	**74.8**	76.9	**68.5**	**72.7**	**0.715**	**0.456**	**0.787**

### Comparison With Other Existing Methods

To further evaluate the generalization of our models, the predictive results of our models on the independent test sets were compared with other existing methods, iRNA-m5C (Lv et al., 2020), m5CPred-SVM (Chen Xiao et al., 2020), RNAm5Cfinder (Li et al., 2018), iRNAm5C-PseDNC (Qiu et al., 2017), RNAm5CPred (Fang et al., 2019), PEA-m5C (Song et al., 2018), and Staem5 (Chai et al., 2021b). However, not all of these methods can predict m5C sites in all three species. For example, RNAm5Cfinder (Li et al., 2018) can predict m5C sites for *H. sapiens* and *M. musculus* but not for *A. thaliana*. iRNAm5C-PseDNC (Qiu et al., 2017) and RNAm5CPred (Fang et al., 2019) can only predict the m5C sites of *H. sapiens*, and PEA-m5C (Song et al., 2018) can only be used for prediction of *A. thaliana*. By using the default decision threshold, Table 6 shows that our model achieved the highest performance for all seven evaluation metrics except specificity for *H. sapiens* compared with other state-of-the-art methods*.* For *M. musculus,* our model obtained the best AUROC, MCC, accuracy, and FOR (false omission rate). For A*. thaliana,* our model achieved the highest values for all seven evaluation metrics. Thus, we prove the superiority of our m5Cpred_XS model for predicting the m5C sites for three species. By using other decision thresholds as shown in Table 6, the precisions, specificities, accuracies, and MCCs of our models can be improved; however, other evaluation metrics, such as sensitivities and F1 scores drop away.

**TABLE 6:** Comparison with other existing models on the independent test sets.

Species	Model^a^	Pre (%)	FOR (%)^b^	Sp (%)	Sn (%)	Acc (%)	F1	Mcc	AUC
*H. sapiens*	RNAm5Cfinder	76.5	41.3	88.4	37.7	63.1	0.505	0.303	0.635
	iRNA-m5C	43.9	55.5	46.4	42.1	44.2	0.429	-0.116	–
	iRNAm5C-PseDNC	60.1	**49.6**	**97.1**	4.4	50.7	0.081	0.039	–
	RNAm5CPred	68.1	30.3	66.7	71.0	68.9	0.695	0.377	0.772
	m5CPred-SVM	78.8	23.6	79.7	75.4	77.5	0.770	0.551	0.858
	Our method (Threshold = 0.5)	80.6	21.1	81.2	**78.3**	79.7	**0.794**	0.594	**0.885**
	Our method (FPR ≈ 10%)	**0.875**	24.4	89.9	71.0	**80.4**	0.784	**0.620**	**0.885**
*M. musculus*	RNAm5Cfinder	64.5	43.8	78.9	38.6	58.8	0.483	0.191	0.593
	iRNA-m5C	**75.1**	49.9	**99.8**	0.6	50.2	0.012	0.032	–
	m5CPred-SVM	73.0	30.0	74.9	**67.9**	71.4	0.704	0.429	0.775
	Staem5	69.7	30.3	77.8	66.1	71.9	**0.735**	0.442	0.787
	Our method (Threshold = 0.5)	74.3	29.9	76.8	67.2	72.0	0.706	0.442	**0.790**
	Our method (FPR = 15%)	79.9	32.3	85.0	59.5	**72.3**	0.682	**0.460**	0.790
*A. thaliana*	iRNA-m5C	73.5	26.7	75.6	72.4	74.1	0.729	0.481	–
	PEA-m5C	43.8	55.6	45.4	43.2	44.3	0.454	-0.114	–
	m5CPred-SVM	76.0	24.4	76.1	75.5	75.8	0.757	0.516	0.836
	Staem5	74.2	25.8	72.6	74.8	73.7	0.734	0.474	0.829
	Our method (Threshold = 0.5)	**77.1**	23.6	77.4	**76.1**	76.8	**0.766**	0.535	**0.838**
	Our method (FPR = 20%)	78.8	24.2	**80.0**	74.4	**77.2**	0.765	**0.545**	**0.838**

a The settings in the parentheses mean different decision thresholds for determining positive prediction.

b FOR, means false omission rate and FOR = FN/(FN + TN).

It is noted that the predictive accuracies of iRNA-5mC and PEA-m5C on the independent test sets are even less than 0.50. The possible reason is that the corresponding training data sets for building these models are small. For example, the model of iRNA-m5C for homo sapiens is based on a data set that only contains 120 positive samples, and PEA-m5C is based on a data set that contains 1196 positive samples. Both data sets were smaller than the data sets used in this study. The small size of the data set limits the generalization of the model on the independent test set. In addition, the model was not evaluated on an independent test set in the original paper of iRNA-m5C and the redundancy of the data set used for PEA-m5C was not removed.

### Implementation of the m5CPred-XS Web Server

To facilitate the use of our model, we built a web server that is freely available at http://m5cpred-xs.zhulab.org.cn/. The server was implemented using flask, docker, and nginx. The users can easily carry out the prediction by the following procedures: First, users can type the query RNA sequences into the input box or upload a FASTA format file. (Note that the input sequence should be in FASTA format, and the length of each query sequence should be longer than 41 bp.) After that, one of the three species, *H. sapiens*, *M. musculus*, and *A. thaliana*, should be chosen. Users can provide their email address as a way to obtain the query results. Then, by clicking the “submit” button, the server generates a unique task ID and do the calculation until the final result is reached. During this process, you can query the task status by task ID. When the task was done, the results would be sent back to the users as an email attachment.

## Discussions

### Analysis of Features Selected by SHAP

**FIGURE 4:**
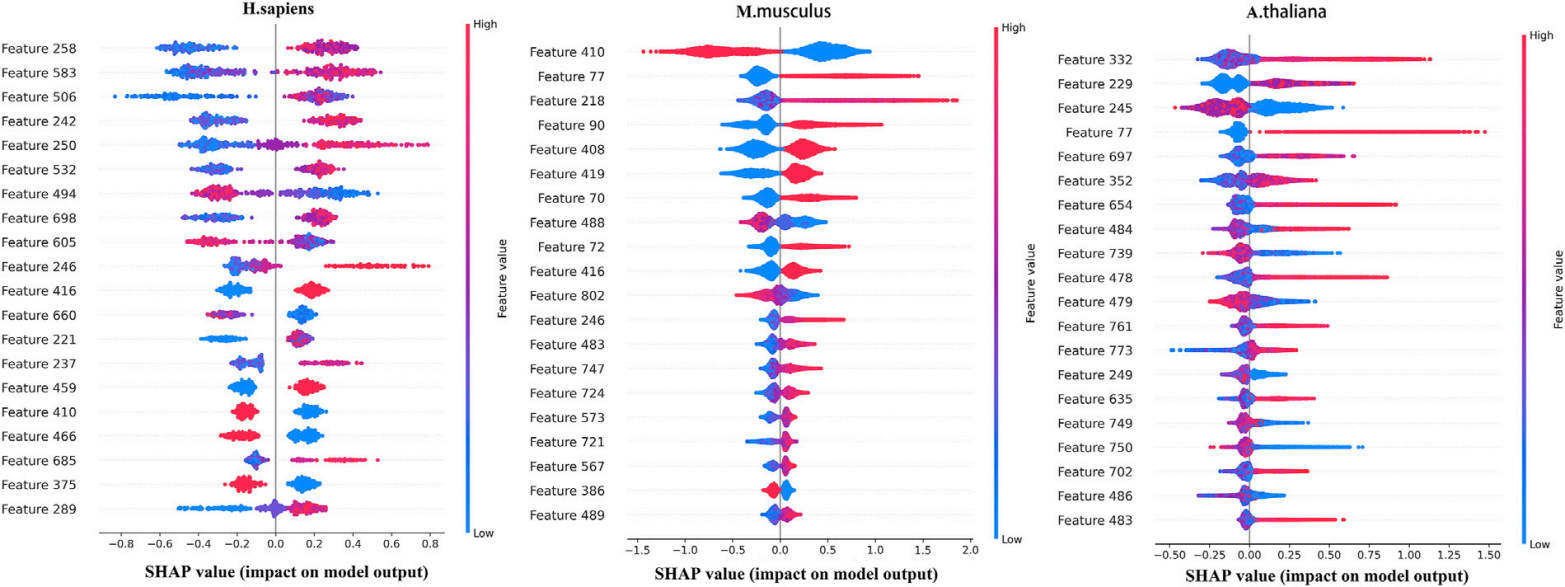
Top 20 features sorted by SHAP for the three species.

To further analyze the features selected by SHAP, the most important top 20 features for the three species are shown in Figure 4, in which the horizontal axis shows the distribution of the SHAP values and the vertical axis shows the features. If the SHAP values are positive, it will help to predict the m5C sites. Otherwise, it means the prediction tends to be of the negative class.

**FIGURE 5:**
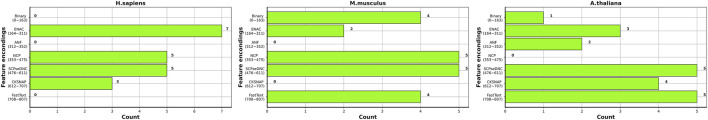
Distribution of top 20 features in the seven types of features for the three species.

Figure 5 shows the distribution of the top 20 features in the seven types of features for three species. Overall, the top 20 most important features are not evenly distributed in the seven types of features for the three species. ENAC and SCPseDNC are the two types of features that appear in the top 20 features of all three species. ENAC represents the detailed distribution of nucleotides in each slide window. SCPseDNC represents the detailed distribution of dinucleotides and the distribution of its physical–chemical properties. Our results indicate that the distribution of nucleotides and their properties are related to the modification. Specifically, when identifying m5C sites of *H. sapiens*, features belonging to ENAC account for the largest proportion of the top 20 most important features, including a total of seven features. The three types of features, binary, ANF, and word2vec, are not included in the top 20 most important features, which indicates that these features contribute little to the prediction m5C sites of *H. sapiens*. For *M. musculus*, five features from NCP and SCPseDNC appeared in the top 20 features, and ANF and CKSNAP did not appear. For *A. thaliana*, five features of SCPseDNC and FastText appeared in top 20 features, and NCP was not included. These results indicate that the relevant features are related to the data sets, and feature selection is helpful for building high-performance models.

**FIGURE 6:**
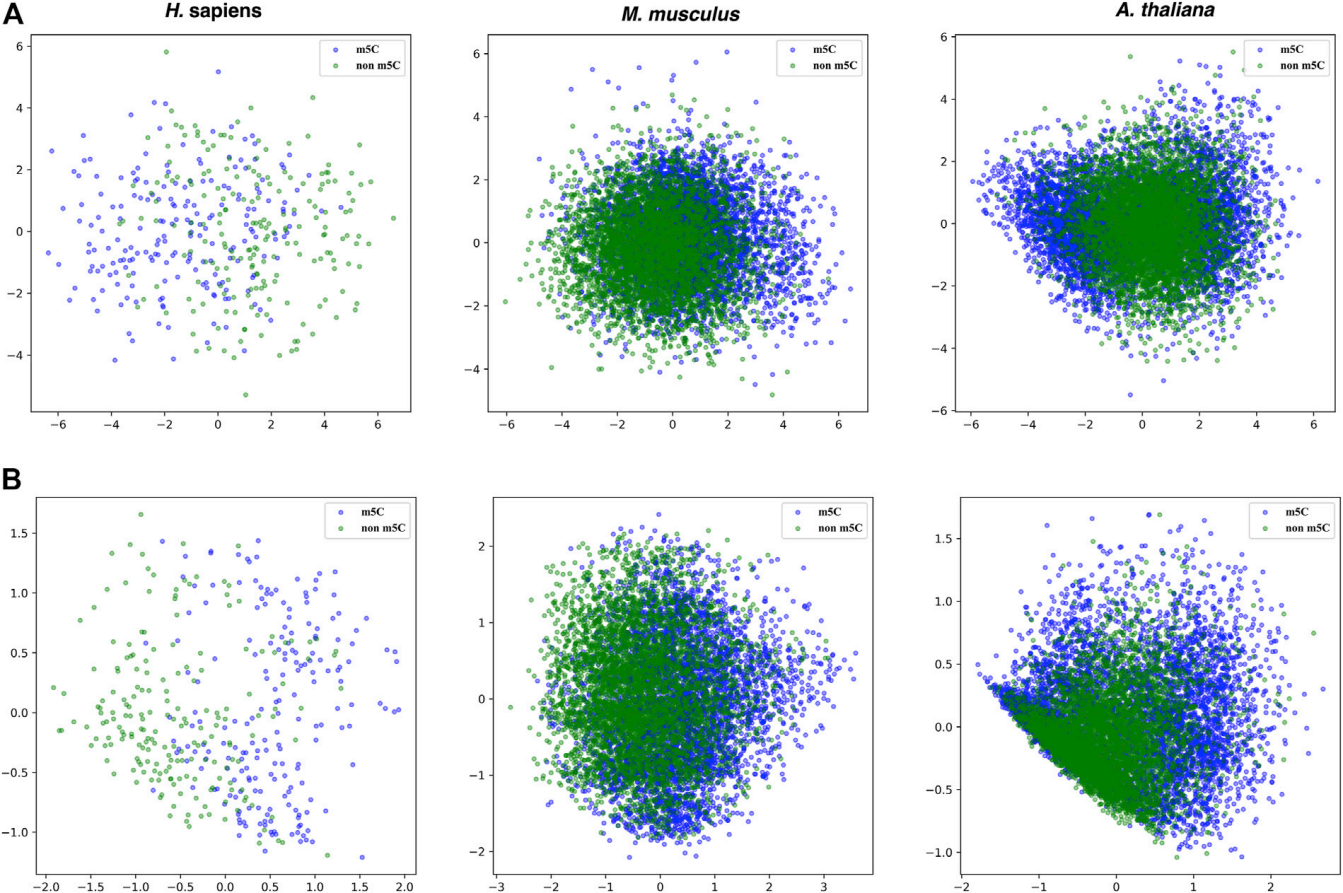
PCA plots for the original 808 dimensional features and features selected by SHAP for the three species. Upper panel: the original 808 dimensional features; Lower panel: the features selected by SHAP.

Moreover, the principal component analysis was used to visualize the effectiveness of the selected features. Figure 6 shows that the boundaries between positive and negative samples for the three species are a little bit clearer in the features selected by SHAP than the original 808 dimensional features.

### Cross-Species Validation

**FIGURE 7:**
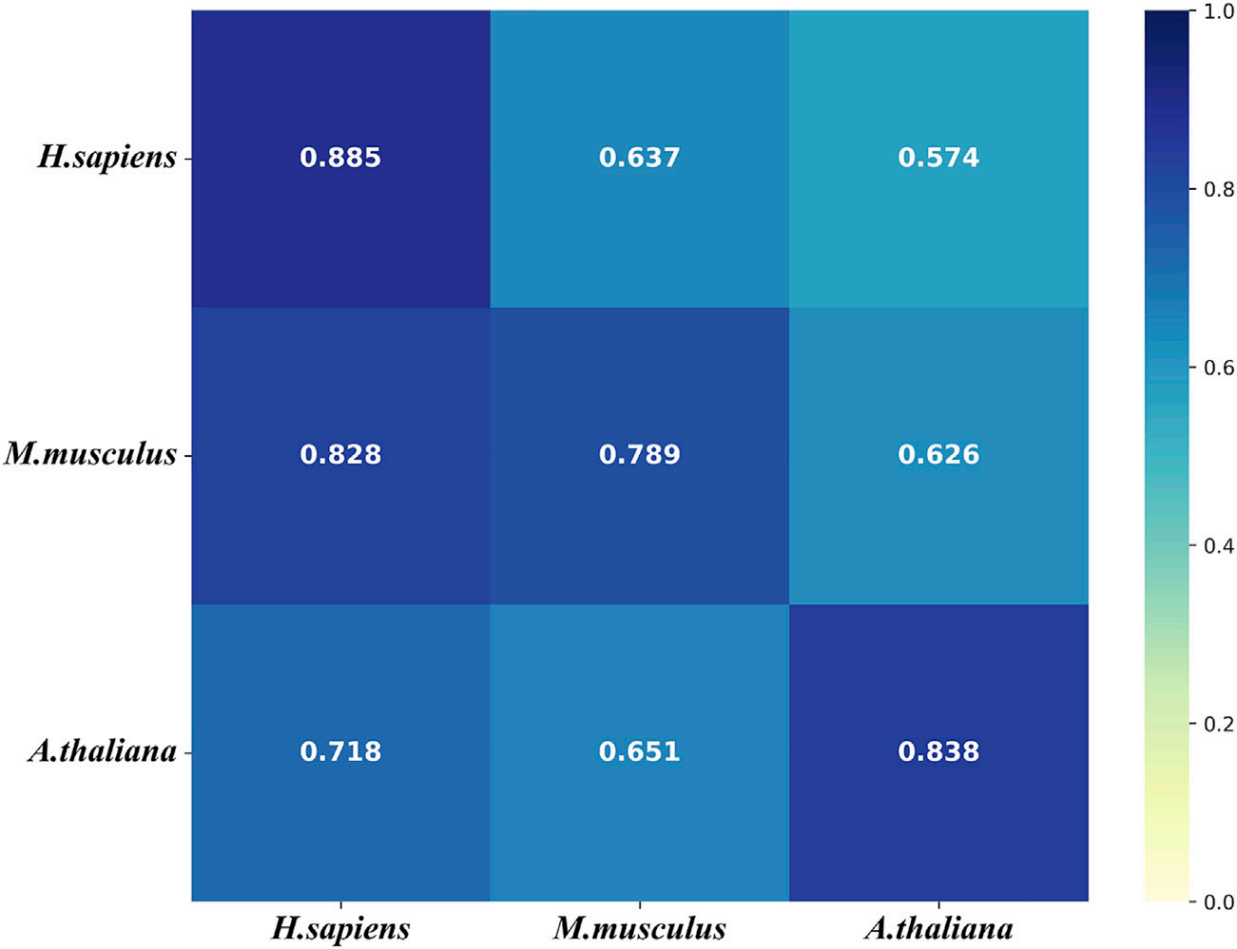
The heat map for the cross species predictive AUROCs. The models (*y*-axis) were tested on the three independent test sets (*x*-axis).

To further evaluate the generalization of our models, we conducted the cross-species validation to analyze the species-specificity and transferability of the models that were tested on the three independent test sets of the three species. Figure 7 shows that the models of all three species performs well (AUROC>0.7) on the independent test set of *H. sapiens*. However, the model of *H. sapiens* does not performs well on the independent test sets of the other two species. Figure 7 also shows that the model of *M. musculus* performs on the independent set of *H. sapiens* even better than that of *M. musculus.* In addition, the model of *A. thaliana* performs worse on the independent test set of *M. musculus*. We thought the small size of the benchmark data set of *H. sapiens* was one of the possible reasons for the results. The other reason is that both *M. musculus* and *H. sapiens* are mammals.

## Conclusion

In this study, we proposed a new computational model, m5Cpred_XS, for predicting m5C sites. Three different feature-selection methods were used to select the optimal subset from 808 dimensional data of seven kinds of features. It turns out that the features selected by SHAP are more relevant compared with the features selected by the other two methods. The selected feature subsets were used to build our models. Our results show that the models based on XGBoost are superior to the models trained with RF and SVM. The m5Cpred_XS was further compared with other existing methods on the independent test sets, which demonstrates that our model outperforms the other methods according to AUROC values.

## Data Availability

Publicly available data sets were analyzed in this study. This data can be available at: https://github.com/yinboliu-git/m5Cpred-XS.
